# Towards improved genome-scale metabolic network reconstructions: unification, transcript specificity and beyond

**DOI:** 10.1093/bib/bbv100

**Published:** 2015-11-28

**Authors:** Thomas Pfau, Maria Pires Pacheco, Thomas Sauter

**Keywords:** metabolic network reconstruction, gene-protein-reaction association, unification

## Abstract

Genome-scale metabolic network reconstructions provide a basis for the investigation of the metabolic properties of an organism. There are reconstructions available for multiple organisms, from prokaryotes to higher organisms and methods for the analysis of a reconstruction. One example is the use of flux balance analysis to improve the yields of a target chemical, which has been applied successfully. However, comparison of results between existing reconstructions and models presents a challenge because of the heterogeneity of the available reconstructions, for example, of standards for presenting gene-protein-reaction associations, nomenclature of metabolites and reactions or selection of protonation states. The lack of comparability for gene identifiers or model-specific reactions without annotated evidence often leads to the creation of a new model from scratch, as data cannot be properly matched otherwise. In this contribution, we propose to improve the predictive power of metabolic models by switching from gene-protein-reaction associations to transcript-isoform-reaction associations, thus taking advantage of the improvement of precision in gene expression measurements. To achieve this precision, we discuss available databases that can be used to retrieve this type of information and point at issues that can arise from their neglect. Further, we stress issues that arise from non-standardized building pipelines, like inconsistencies in protonation states. In addition, problems arising from the use of non-specific cofactors, e.g. artificial futile cycles, are discussed, and finally efforts of the metabolic modelling community to unify model reconstructions are highlighted.

## Introduction

Over the past two decades, the increasing availability of genomic, proteomic and metabolomic information has led to the generation of a multitude of metabolic network reconstructions [1]. These reconstructions aim to represent our collective knowledge about the metabolism of the reconstructed organisms. They serve as a source of information on their target organism, and models derived from the reconstructions can be used to investigate its metabolic capabilities. The available reconstructions cover multiple types of organisms, ranging from microorganisms, like *Escherichia coli* [2–4] and *Saccharomyces cerevisiae* [5, 6], to complex multicellular organisms, like *Arabidopsis thaliana* [7–9] or *Homo sapiens* [10–12].

Despite the availability of high-quality protocols for the reconstruction of a genome-wide network [13], efforts are far from consistent between different groups. The most common differences are multiple naming schemes for reactions, metabolites and genes, along with different formats for reconstruction exchange. Some of the issues arising from these differences have been discussed in Monk *et al.* [14]. The main challenge is to compare networks generated by different reconstruction tools, or using different naming schemes [15]. Furthermore, the lack of precise annotations leads to information being overlooked, which could improve the models resulting from reconstruction efforts. With automation of model generation [16, 17], in particular towards tissue-specific sub-models [18, 19], it becomes ever more important that reconstructions are curated in a consistent way.

There have been attempts to establish databases that can help in generating consistent networks by providing links to multiple databases, like MetRxn or MetaNetX [15, 20]. These studies also highlighted the issues arising from the multitude of naming schemes used. While we know that there are multiple pathways that are shared between a multitude of organisms (like glycolysis or the Krebs cycle), finding these similarities in reconstructions is challenging. The authors of MetRxn report that by using simple string matching techniques only three reactions could be directly inferred as being identical in a set of over 30 models [15]. Thus, unification is paramount to determine the novelty of new reconstructions.

Unified representation, however, is not the only issue with current reconstructions. Most reconstructions rely purely on genetic information for functional annotation; however, recent advances in both microarray and RNA-seq technologies provide information about messenger RNA (mRNA) on a transcript level. Inclusion of this kind of information could potentially increase the accuracy of models. Another issue that can influence predictions is cofactor specificity, which has been shown to be influential in metabolic modelling [21]. In this article, we will highlight potential approaches to unify metabolic network representations and highlight the importance of transcript specificity to metabolic networks. We will further elaborate on the issues arising from cofactor specificity in metabolic network analysis (e.g. sets of reactions using either NADPH or NADH, which can form futile cycles, indicating those reactions as active while in truth they are disconnected from the network). Finally, we will provide an overview of projects aiming at improving the current lack of unification, by coordinating multiple reconstruction efforts for the same organism, or creating databases with compatible networks.

## Steps towards a unification of model representation

Metabolites and reactions linking them form the core of a metabolic network. Additional information is often provided in the form of genes that code for enzymes catalyzing a specific reaction. These can be simply lists of genes associated with a reaction, or they can form gene-protein-reaction (GPR) association rules representing protein complex formation. To provide this information, multiple different types of formats have been used (see Table 1). Some, like the Systems Biology Markup Language (SBML, [32]) or spreadsheets, are platform independent, while others, like MATLAB structs, depend on a specific software. The advantage of SBML over other formats is its versatility, and general usability by almost all current software tools specific to metabolic modelling (for recent reviews on these tools, see Lakshmanan *et al.* [33] or Dandekar *et al.* [34]). Nowadays, most models are indeed published in the SBML format [25, 35–37]. In addition, many software tools, even if they have an alternative internal storage format, like ScrumPy [27], COBRA [22], RAVEN [17] or Pathway Tools [38], provide some type of import and export functionality to read and generate SBML files that can be used as input into other tools. However, there are still models like the latest versions of the popular metabolic network reconstruction of *H. sapiens*, Recon2, which are only available as a MATLAB export specific to the COBRA toolbox environment [22]. Beyond the common general file format, models tend to diverge substantially.

**Table 1:** Different formats for the exchange of metabolic models

Model style	Description	Advantages//Disadvantages	Examples
SBML/COBRA	SBML with additional information in the notes sections of entries [22]	Models are usable in any SBML capable tool, but the additional information needs explicit parsers. Tool independent.//There is no clear definition of used fields in the SBML format, and different groups use multiple different data fields.	BiGG models [23], MetaCyc SBMLs [24], iJO1366 [3]
SBML/Mod	SBML using ‘ModifierSpecies' to define GPRs	Models are usable in any SBML capable tool. Genes can be linked to multiple sources. Proteins can be encoded and linked explicitly. Tool independent.//Needs parsers that make use of these properties. Lacks a defined standard how ‘ModifierSpecies' have to be defined.	HMR [25], yeast consensus [6]
SBML/FBC	SBML with FBC extension for FBA-specific information [26]	Uses SBML defined fields (from the FBC extension) to provide FBA-specific information. Proteins can be encoded (and identified) explicitly. Tool independent.//FBC extension not yet processed by many tools.	BiGG2 Database (http://bigg.ucsd.edu)
Toolbox-specific formats	Formats specific to one modelling tool, e.g. COBRA MATLAB files [22] or ScrumPy.spy files [27]	Files can directly be used in the respective toolbox and can contain additional information.//Not easily loaded into other tools.	Recon2 [12], iMM1415 [28] Poolman *et al.* [7]
Spread sheets	Commonly multiple sheets or files with compounds, reactions and genes	Easily accessible for non-computational users. Tool independent.//Difficult to parse for further analysis, because of the lack of a standard format.	HepatoNet [29], Oh *et al.* [30], iNJ661 [31]

*Note*. Annotation of the SBML is either achieved by COBRA notes fields (e.g. for Database links), or using BQ and the annotation class of SBML. Both types have been used in combination with SBML/Mod and SBML/COBRA, even though commonly SBML/COBRA models do not include BQ annotations, as they rely on the COBRA annotations.

### Flux balance-specific information

GPR association rules, which are commonly used to link gene expression or proteomics data to metabolic networks [39–42], are inconsistently represented in different models. While some reconstructions provide those GPRs in supplemental spreadsheets [29], the COBRA toolbox defines additional fields in the SBML ‘Notes' section of a reaction, which contain the GPR rules [22]. Recently some reconstructions, like the yeast consensus model [6] or the Human Metabolic Reconstruction (HMR) [25], provide ‘ModifierSpecies', which are annotated as being encoded by specific genes using bio-qualifiers [43]. The COBRA toolbox also added further information into the ‘Notes' section, including metabolite formula and charge information, or information on pathways that include a given reaction. While this information is useful for network analysis, it lacks a clear definition of which fields can be used or should be present. Thus, multiple different fields have been used across models, with some fields remaining undefined in some models. However, this information could also be provided within the annotation field of a metabolite or reaction using biomodel qualifiers (BQ) [43], e.g. a reaction ‘isPartOf' a specific pathway, without the necessity of additional field definition. Another specification made by the COBRA toolbox was to use the ‘kineticLaw' field to define flux constraints, thus using a structure that is not designed to hold this information but is supposed to be used for real kinetic information. Because SBML is a general systems biology representation, this could lead to confusion if the structure of a stoichiometric model is imported into a kinetic tool. These inconsistencies in the use of SBML, in addition to the increasing amount of available reconstructions, have prompted the development of the flux balance constraints (FBC) extension [44] to SBML, which covers many aspects specific to flux balance analysis (FBA). While initially only providing support for flux bounds and providing additional SBML fields for charge and formula within the ‘Species' class, the latest version (Version2, Release 1 [26]) also provides facilities to handle GPRs, including the option to add gene products (thus directly adding protein identifiers to the model along with gene/transcript identifiers). FBC further allows the inclusion of specific settings for simulations in ‘FluxObjectives'. The clear definition of the FBC extension along with its direct link to the SBML specification makes it an ideal choice for data provision.

### Naming conventions and comparability

While the ‘FBC’ extension handles many of the aspects specific to flux balance models, there is still wide diversity in naming schemes used for metabolite or reaction identification and the choice of gene representation. Until now, there are no generally accepted naming conventions for metabolites or reactions, and thus the choice of identifiers strongly depends on the database used as a basis for the reconstruction, or how the researchers choose to define their system. Naming schemes have included custom abbreviations [45, 46], consecutive numberings [29] or extracted identifiers from databases [7].

Newer reconstructions tend to make extensive use of the SBML annotation field, Systems Biology Ontology identifiers (see http://www.ebi.ac.uk/sbo/) and BQs. Usage of these qualifiers in addition to adherence to standards defined as the ‘Minimum Information Required In the Annotation of Models' (MIRIAM) [47] will make it possible to create universally applicable interpreters and tools. However, even when trying to adhere to the MIRIAM standards, it is important to select a proper set of resources to annotate the model components. There are multiple databases for compounds (e.g. CHEBI [48], PubChem [49], KEGG [50], MetaCyc [24]), reactions (KEGG, MetaCyc, BRENDA [51], GO [52]), proteins (BRENDA, UniProt [53], PDB [54], ENZYME [55]) and genes (NCBI—Gene [56], UniProt, GeneDB [57], GeneCards [58]), with some (like KEGG and MetaCyc) catering primarily to metabolism, while others are more comprehensive.

As new models are commonly accompanied by novel functionalities or entities, databases that allow the deposition of new entries would be preferable. While the most popular metabolic databases (MetaCyc and KEGG) do contain entry types on the most relevant entities, they do not allow a direct deposition of new entries. They are therefore unsuitable for deposition of newly developed models, as this would lead to new identifiers that cannot be directly used by others. Using multiple databases to solve this issue can introduce new sources of errors. For metabolites, one database might consider all compounds to be present at a certain pH (like MetaCyc), while other databases represent the same compound as fully protonated (like BRENDA). Thus, when trying to determine charge balance or hydrogen balance, issues arise if inconsistent sources are used, and one source might not provide the required protonation state for all compounds in the reconstruction. If novel compounds, proteins or genes are introduced in a reconstruction, we would recommend using CHEBI, UniProt and NCBI—Gene to directly deposit the novel entries and use them to annotate the entities in the model. For known compounds, a selection of consistent sources (e.g. the same protonation state as in the reconstruction) would, in our opinion, be more suitable than a large selection of databases, with different definitions, to avoid confusion.

## Transcripts—the information lost in reconstructions

As mentioned above, GPRs are informationally important in metabolic reconstructions, in particular when trying to integrate omics data into metabolic networks, e.g. to extract context-specific models from a generic genome-wide reconstruction. The GPRs annotated in metabolic reconstructions mostly consider only genes, completely neglecting the fact that one locus can be translated in different variants through alternative splicing.

**Figure 1:**
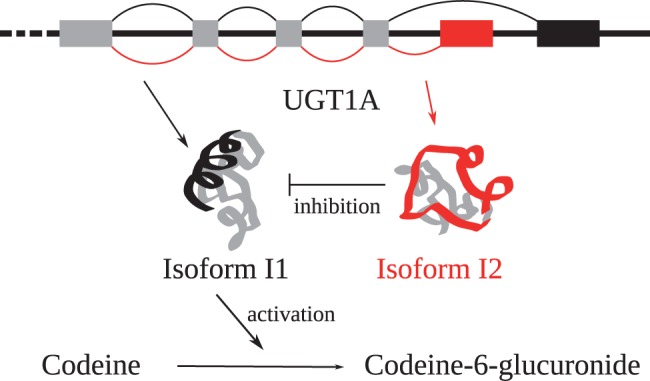
Alternative splice forms are created by removal and addition of exons during the splicing process. This example shows two the alternate splice forms i1 (depicted in black) and i2 (depicted in red) of a human glucuronosyltransferase (UGT1A). The main isoforme, i1, is implicated in the metabolism and excretion of toxic compounds, e.g. drugs like codeine, while isoform i2 inhibits the activity of the main isoform. A colour version of this figure is available online at BIB online: https://academic.oup.com/bib.

Alternative splicing (as shown in Figure 1) allows increased diversity and regulatory complexity of an organism without requiring a massive increase in genome size [59]. It is particularly important in humans, with splicing variants affecting 95% of the genes [60, 61]. Even if the different variants have mostly similar functions, in some cases, the alternative variants have opposing effects, like the caspase 8 isoforms that are anti- and pro-apoptotic [62]; provide insufficient activity, as in the instance of the TAZ gene [63]; or inhibit the main isoform. An example for the latter is isoform i2 of UGT1A that negatively modulates the glucuronosyltransferase activity of isoform i1 [64, 65].

In general, several splice variants are simultaneously expressed, although usually one variant dominates the others, accounting for on average 85% of the protein-coding mRNA at a given loci [66]. The dominant variant is usually highly conserved during evolution, but the expression pattern is constantly changing to meet cell- and condition-specific requirements [67]. Not only do different cell types have a different set of variants, but also different individuals show different splicing. Furthermore, switch-like effects, where variants lose their dominant position in favour of other variants, were observed for hundreds of genes during differentiation [68, 69], demonstrating the plasticity of a tightly regulated process. Alterations of the latter are implicated in numerous pathologies, especially in cancer, and several splice variants are even considered as biomarkers, like PRKC-*ζ*-PrC for prostate cancer, Nek2C for breast cancer and CD-44 splice variants for colon cancer [70–72].

### Current use of transcripts

Most metabolic models do not consider transcript variants, as functional information is often only available at the gene or protein level. Even metabolic reconstructions that introduced transcript identifiers in their GPR association rules based on bibliographic research, like Recon1 [10], do not allow mapping of the transcripts identifiers of the model to transcript identifiers used by databases. This issue arises from the lack of direct matching between the reconstruction identifier and available databases’ identifiers. Therefore, in practice, the information related to splicing variants is simply ignored. GPRs are gene oriented, and, as a consequence, the intensity levels of the transcripts variants are usually simply summed up or the maximal intensity values are mapped to the reactions of the model.

Alternative splicing was shown to be altered in a wide range of diseases [73, 74]. In cancer, usually minor isoforms get overexpressed and dominate the main splice form. For example, the alternative splice form pyruvate kinase isoform 2 (PKM2) favours aerobic glycolysis, whereas the main form promotes oxidative phosphorylation. The expression of PKM2, which is the embryonic isoform, is restricted in adults to cancer cells that do not express PKM1 [75]. A model with gene-oriented GPRs cannot differentiate between the two isoforms and will therefore consider the same set of target reactions as active for both isoforms.

The existence of tissue- or context-specific alternative exons involved in the same pathways, and regulated by common mechanisms as, for example, the neural-specific splicing regulator nSR100/SRRM4, was demonstrated in several studies [76–80]. Although alternative exons were mostly studied for their impact on protein–protein interaction networks, it is probable that alternative exons have a similar role in metabolic modelling, controlling the activation of tissue-specific metabolic sub-pathways. In this case, a model with gene-oriented GPRs would fail to capture tissue-specific activation patterns.

### Sources for transcript-specific information

The prevalent barrier to the inclusion of transcript variants in metabolic network reconstruction is the lack of knowledge about the alternative splice forms in most organisms. Databases collecting information on alternative splicing are mainly dedicated to humans, mice and other vertebrates because splicing is most important in eukaryotic organisms. The largest benefit of this endeavour is therefore expected for human models, where the inclusion of transcript information could explain pathologies linked to alternative splice forms, e.g. in cancer [81, 82], neurodegenerative diseases [83–85] or autosomal dominant retinitis pigmentosum [86]. The inclusion of information on alternative splice forms will increase the capacity of cell-specific and context-specific models to capture the variability in metabolism of different cell types. However, even for the organisms with the highest information content on alternative splicing, the functional activity of most splice forms remains unknown. To address this problem, several databases have been dedicated for a decade to collecting transcript information. These include ASAP II [87], ECGene [88], ASTD [89], HOLLYWOOD [90], H-DBAS [91], FAST DB [92] and FANTOM 3 [93], which try to supplement generic gene databases (ENSEMBL [94, 95], Pfam [96, 97], Uniprot/Swiss-Prot [98]). A more intensive review of these databases can be found in Kelemen *et al.* [99] or Taneri and Gaasterland [100].

#### Problems of automated annotation pipelines

The increased amount of data on alternative splicing obtained through deep-sequencing technologies outpaces the capacity of databases to completely annotate the transcripts manually, and therefore nearly all databases use semi-automated or automated pipelines. Automated annotation process are more prone to errors than manual curation. The rate of wrong annotation in GenBank [101], NR [102], TrEMBL [98] and KEGG [103] was assessed by Schnoes *et al.* [104], who tested 37 enzyme families. They found misannotation rates ranging from 5% up to 63% for the automated databases, whereas Swissprot, which performs manual curation, had a misannotation rate close to 0 [104]. A similar misannotation rate because of the automated pipeline is expected for alternative splice forms. Several strategies can be used to identify the function of a new alternative splice form. The most common compares the sequence of the transcripts or the isoforms to species already present in the databases using tools like Basic Local Alignment Search Tool. The reliability of the annotation depends equally on the quality of the algorithms uses and the correctness of the annotations of species already present in the databases. Although algorithms do create errors in the identification of open reading frames, the database entries themselves might be more problematic, as erroneous entries can propagate quickly through automated methods. For example, one of the most used databases [105], GenBank, only allows the sequence submitter to correct or update the submitted annotation. This leads to few corrections and updates, thus accumulating errors in a database that shares its entries with several other databases [105]. In addition, the prediction of function based on the amino-acid sequence, taking advantage of massive high-throughput data, is getting more popular. The different tools used by the databases have different accuracy levels, and the characteristics of the annotation tools must be taken into consideration when selecting a reference database.

#### Transcript databases suitable for metabolic model annotation

The GENCODE collaboration [106] tries to annotate genes and splice variants discovered by the ENCODE consortium [107] using a combination of manual curation, automated annotation pipelines and targeted validation approaches. Within the GENCODE collaboration, the APPRIS database [66] is dedicated to the annotation of principal and alternative splice isoforms. The aim of APPRIS is to validate manually annotated isoforms with functional data and protein structures. APPRIS selects the major isoform that is present in most cells and contexts and compares that isoform to all other isoforms. APPRIS could identify the dominant variants of 85% of the protein-coding transcripts of the GENCODE 7 release for ENSEMBL [94, 95].

Vega [108], a database for vertebrate genomes that contains a section with annotations for alternative splicing information, is another useful source of transcript information. The human and vertebrate analysis and annotation team is actively participating in these annotation efforts, and it was incorporated into the set of ENSEMBL databases [94, 95]. The aim is to systematically annotate all experimentally validated expressed sequence tags or mRNAs from ENCODE [107] and the 1000 Genomes loss-of-function project [109], without prior filtering based, for example, on the tissue of origin. This unbiased approach allows the annotation of transcripts that do not yet have an obvious function.

The ASPicDB database [110] considers the human isoforms that result from alternative splicing events. Annotation is then performed by machine-learning approaches that categorize the proteins by function, localization, transmembrane domains, signal peptides, gpi- and coiled-coil domains, and similarity to known peptide sequences. The ADPicDB database uses the ASPIC algorithm [111] to perform multi-alignments to the genome. The alignment that minimizes the splicing events is then retained.

H-DBAS II [112] is the successor of H-DBAS [91], a database that collects information on human alternative splice forms, with the focus on alternative splicing events altering protein functions. The H-DBAS database was mainly based on cDNA libraries. H-DBAS II now takes advantage of the RNA-seq technology to improve the annotation of splicing variants.

The SASD database [113] predicts alternative splice forms expressed in different contexts, e.g. during disease, under drug effects or in different organs. Data extracted from ENSEMBL [95] and from the Integrated Pathway Analysis database are used to create artificial transcripts and peptides.

While all databases mentioned above are focusing on different vertebrates, the ASIP database is specialized to plants [114]. It allows the visualization of alternative splice forms in plants like *A. thaliana* or *Oryza sativa*. To obtain the annotations, the ASIP database uses an automated approach based on alignment tools.

Table 2 gives an overview of these databases, which, along with further information provided by RNA-seq experiments, represent a valuable source of data that could increase the predictive capabilities of metabolic models. Besides automated pipelines to map the correct transcripts to known metabolic reactions, data mining approaches and bibliographic research similar to those performed by the Recon1 project would be required to unravel the function of the variants. It would, however, be important to use these resources to implement a common nomenclature that would prevent information loss and create consistency between models.

**Table 2:** Databases for transcript-specific genome annotations of multiple species

Name	Species	Method of annotation	Reference	Link
GENCODE	Human and mouse	Manual and automated	[106]	http://www.gencodegenes.org/
ASPicDB	Human	Automated	[110]	http://srv00.ibbe.cnr.it/ASPicDB/
Vega	Human, zebrafish, pig, mouse and rat	Manual annotation	[108]	http://vega.sanger.ac.uk/index.html
H-DBAS	human, mouse, rat, chimpanzee, macaque and dog	Manual	[112]	http://www.h-invitational.jp/h-dbas/
SASD	Human	Prediction	[113]	http://bioinfo.hsc.unt.edu/sasd/
ASIP	Plants	Automated	[114]	http://www.plantgdb.org/

## Non-specific cofactors can cause infeasible loops

Another issue commonly observed when reconstructing metabolic networks is the difficulty of selecting the right cofactors for reactions, specifically the right redox pairs. The assignment of cofactors to reactions is complicated by the fact that the cofactor requirement is organism specific and cell specific, explaining at least partially that the cofactors requirements vary between databases [117]. Furthermore, gene matching algorithms used to reconstruct networks will often find reactions using all potential cofactors and include them in the reconstruction. The discrepancies are further accentuated by the fact that in the case of missing electron transfer pair information, NAD^+^/NADH is most often the default transfer cofactor used [116]. The reason for this default choice is that finding organism-specific information is not trivial and can necessitate extensive literature research even for well-studied organisms. Furthermore, several enzymes have different isoforms that do not exhibit the same cofactor requirements. One example is aldehyde dehydrogenases, which may use both NADH and NADPH. In the cytoplasm of *S. cerevisiae*, the main isoform uses NADP^+^, whereas stress-induced isoforms prefer NAD^+^ as cofactor [117]. Unfortunately, databases tend to either provide unspecific reactions [using NAD(P)^+^], only one variant, or often both variants associated with both genes in these instances, which makes it challenging to assign the correct reaction to the respective isoform. In addition, several enzymes are able to catalyze various reactions, and the catalyzed reactions depend on the availability of a specific cofactor. This leads to the incorporation of all potentially catalyzed reactions that vary only by their cofactor requirements [5], which is likely to cause loops or cycles that are thermodynamically infeasible if one or more of the reactions are reversible. Loops carry a non-zero flux, even in the absence of an input and output flux, if no thermodynamical constraints are added. These loops violate the loop law, a law similar to Kirchhoff’s second law for electrical circuits. There have been attempts to eliminate the presence of thermodynamically infeasible loops from FBA calculations and it has been shown that their presence can diminish the predictive power of models [23]. However, the use of loopless FBA converts the simple linear problem into a mixed integer linear problem, which can lead to long computational times, particularly if multiple rounds of the problem have to be solved. Other approaches to solving this issue show similar characteristics with respect to computational requirements [118] and are therefore often not included in the analysis of metabolic models.

## Community efforts to improve metabolic models

There have been attempts to create collections of metabolic networks, e.g. Model SEED [16] or BiGG [23], and unify identifiers like MetRxn [15] or MetaNetX [20] (listed in Table 3).

**Table 3:** Databases aiming at providing functional metabolic models that are directly comparable

Resource	Unification	Description
BiGG [23]	SBML/COBRA	Database containing multiple genome-scale metabolic networks in the COBRA format.
BiGG2 (http://bigg.ucsd.edu)	SBML/COBRA, SBML/FBC	Update to BiGG, currently in a beta version, providing multiple models annotated using FBC.
MetaCyc [24]	SBML/COBRA, biocyc flat files	Large collection of metabolic reconstructions. Flat File format contains additional details not included in the provided SBMLs.
SEED [16]	SEED IDs, Partial SBML/COBRA format	System for construction of metabolic reconstructions and analysis. Export of reconstructions is available in SBML format (with minimal annotations) and Excel sheets.
MetaNetX [19]	MNXRef IDs, SBML/COBRA, bioql information for metabolites	Repository of unified metabolic reconstructions linking to multiple external databases. Offers tools for network analysis and modifications. SBML files contain additional yeast-style annotations for species.
MetRxn [15]	MetRxn ID, SBML/COBRA	Database matching multiple metabolite and reaction databases aiming at providing a curated basis for network reconstruction.

Model SEED is aimed at providing a platform for model reconstruction based on automated genome annotation using RAST [119]. While this is sufficient for the analysis tools provided on the website, the exportable model formats lack unification information. They do adhere to the COBRA toolbox standard, but as mentioned earlier, that definition itself lacks a lot of information. BiGG was introduced to allow comparison between different networks, but relied on all deposited networks adhering to the same nomenclature, and is restricted by the limited number of deposited reconstructions. The database is currently being updated however, and a beta version of BiGG2, comprising lots of additional models and providing well annotated models, has recently been made available online.

In contrast to this approach, MetRxn and MetaNetX aim at identifying common reactions by combining multiple pieces of information. Bernard *et al.* [120] give a good overview of the issues arising when trying to match metabolites, and how different databases try to address them. The biggest issues arise from stereoisomers and difference in protonation states. While most often protonation states can be ignored (as long as they are consistent within a model), there might be issues when different compartments exhibit different pH. This could become particularly important for energetic considerations if different protonation states are assumed for mitochondria and cytosol. The same problems can potentially arise from considering equality of stereoisomers, with different stereoisomers being processed at different efficiencies [121]. Both MetRxn and MetaNetX can be a great help to overcome most of these issues, with MetaNetX being the more comprehensive approach. Using an extensive set of external databases, it tries to match similar external compounds to its namespace. To address issues of stereoisomers and protonation states, it provides a distinction between identical structures, structures with the same tautomeric form at pH 7.3 and inferred similarities. Even though this information is not directly visible on the website, it can be retrieved from the data export files. However useful these tools become, it is even more important that they are used, and that the community works in concert to improve models, avoiding the creation of multiple distinct reconstructions for the same organism. While the exchange of models in a common language would be an important step, as it would make the combination of models easier, we also want to highlight two recent collaborative efforts that lead to the development of more comprehensive reconstructions.

The first example of a successful community effort for organism-specific reconstruction is the creation of the consensus model of *S. cerevisiae*. Several models of yeast had been published [122–124] until, in 2007, a combined effort was undertaken to merge these models and bring them into a more standardized format [125]. This early combined effort now led the 7th iteration of the model [6], which inspired the formulation of GPRs as suggested above.

Another example of community effort to merge models is the HMR Recon 2 [12]. The first human genome-scale metabolic reconstruction, HumanCyc, was published in 2005 [126]. Soon after, two refined genome-scale reconstructions were published; Recon 1 by Duarte *et al.* [10], and the Edinburgh Human Metabolic Network (EHMN) by Ma *et al.* [11]. These competing models, along with HepatoNet [29] and further information from the literature, were combined into Recon 2 [12] in an effort to unify the different sources. While the attempt led to a more complete knowledge source, it reinforced the problems of incompatibility between different networks. For example, Recon 1 used Entrez gene identifiers with transcript-specific details as gene IDs, while HepatoNet used gene symbols, leading to mixed identifiers in Recon 2, which makes simulations more challenging. In addition, the transcript-specific information from Recon 1 got mostly lost because it, unfortunately, was not traceable to databases Section 3, and neither EHMN nor HepatoNet contained similar information. This again highlights the importance of linking information to databases because great efforts can be lost or have to be repeated. Still, Recon 2 is an important step in the development of HMRs and is only in its second iteration. There remain competing reconstructions or knowledge bases like the HMR, which will be merged in the future.

Key Points

- The increasing amount of metabolic reconstructions necessitates a more unified way of representation to make models comparable.
- Available unification sources could provide a basis for this process.
- Associations to genetic information in metabolic reconstructions need a clearer and more structured association.
- Transcript-specific association rules would improve the specificity of network activities.
- Cofactor specificity needs to be addressed more carefully during reconstruction.
